# Effect of Nano-SiO_2_/PVA on Corrosion Behavior of Steel Rebar Embedded in High-Volume Fly Ash Mortar under Accelerated Chloride Attack

**DOI:** 10.3390/ma15113900

**Published:** 2022-05-30

**Authors:** Jingjing Huang, Zhongkun Wang, Dongsheng Li, Gengying Li

**Affiliations:** 1 Department of Civil Engineering, Shantou University, Shantou 515063, China; 15jjhuang2@stu.edu.cn (J.H.); lids@stu.edu.cn (D.L.); 2 College of Civil Engineering and Architecture, Wenzhou University, Wenzhou 325035, China; 13526578601@163.com; 3 College of Water Conservancy and Civil Engineering, South China Agricultural University, Guangzhou 510642, China

**Keywords:** nano-SiO_2_, PVA fibers, high-volume fly ash cement mortar, accelerated chloride attack, impressed voltage technique

## Abstract

In this paper, the influence of Nano-silica (NS) and Polyvinyl alcohol (PVA) fibers on the corrosion behavior of steel rebar embedded in high-volume fly ash cement mortars under accelerated chloride attack was studied by using an impressed voltage technique. The PVA fibers used were 1.0 vol.%, and two mass fractions of cement (50 and 60 wt.%) were replaced by fly ash. Four NS mass fractions (0, 0.5, 1.0, and 1.5 wt.%) were utilized in this paper. In addition, the mono and hybrid effects of NS and PVA on the mechanical properties and water absorption of mortar were also studied. The results showed that the incorporation of PVA and nano-SiO_2_ can improve the flexural and compressive strengths of high-volume fly ash mortar. Generally, the flexural and compressive strengths increased with the increase of nano-SiO_2_ content. Moreover, the incorporation NS can also reduce the capillary water–absorption rate of cement mortar. The impressed voltage corrosion test indicated that the composite incorporation of nano-SiO_2_ and PVA can significantly delay the deterioration process of steel bars in mortar, effectively reducing the steel rebar’s corrosion level and increasing the exposure time of the surface crack. With hybrid-incorporation 1.0 vol.% PVA and 1.0 wt.% nano-SiO_2_, the steel rebar had the lowest corrosion degree, which exhibited a mass loss of 49% and increased the broken time by 71% as compared to the control mortar.

## 1. Introduction

Reinforced concrete is currently one of the most widely used materials in the field of civil engineering, mainly because it is convenient to obtain and cheap in cost. However, the massive use of cement in reinforced concrete has a negative impact on the environment as the production of cement consumes a lot of energy and natural resources, accompanied by the release of CO_2_ and dust particles. Fly ash, a kind of industrial solid waste, not only has pore-filling and morphology effects but also can react with Ca(OH)_2_ (one of the products of cement hydration production) to form C-S-H and Aft, leading to the enhancement in the microstructure and durability of concrete [1,2]. Moreover, the replacement if cement with fly ash (especially using high-volume fly ash) can reduce the negative effects of stacking fly ash waste and producing cement. Furthermore, the use of a high volume of fly ash can greatly decrease the cost of concrete as fly ash is much cheaper than cement. Thus, the use of a high volume of fly ash in concrete has good economic and social benefits. However, the substitute of cement by high-volume fly ash may decrease the pH value of concrete as the secondary hydration of fly ash needs to consume Ca(OH)_2_. According to Jing Yu et al. [2], the replacing of 40% fly ash could decrease the Ca(OH)_2_ content from 6.5% to 3.0% after 360 days curing. The increase in fly ash replacement would further decrease the Ca(OH)_2_ content, where the replacing of 60%, 80%, and 98% fly ash, after 360-days curing, decreased the Ca(OH)_2_ content from 6.5% to 2.1%, 0.2%, and 0.1%, respectively. This heavy consumption of Ca(OH)_2_ may have a negative influence on the corrosion behavior of steel rebar in reinforced concrete. It is well known that the internal environment of concrete presents a high-alkaline environment with a pH value of 10.5–13.5. In this high-alkaline environment, the steel rebar’s surface forms a layer of passive film, which can effectively prevent the steel rebar from corroding [3]. However, when harmful media (carbon dioxide, chloride, sulfate, etc.) penetrate into the concrete, the internal environment pH drops and the passive film of the steel rebar may be damaged [4,5], resulting in corrosion of the steel rebar. Rust volume may be five times larger than that of un-corroded steel, so internal cracks may occur. Harmful media in the environment directly penetrate into the concrete through the cracks, accelerating the corrosion of steel rebar, which seriously affects the safety and durability of the concrete structure and reduces the service life of the reinforced concrete structure [6,7,8].

The corrosion of steel rebar is an electrochemical process in the concrete. At the anode, iron is oxidized to form iron ions, while at the cathode, oxygen and water are reduced to OH^−^ and enter solution. In this way, the anode and cathode form a corrosion battery, because of the flow of ions and electrons in the concrete. The compactness of the concrete affects the corrosion rate of steel rebar. The higher the density of concrete, the more difficult it is for oxygen and moisture to enter the interior of the concrete, and the transmission speed of the ions also slows down, eventually leading to a reduction in the steel rebar’s corrosion rate. The electrical conductivity of concrete, the nature of the steel–concrete interface, and the thickness and quality of the concrete cover also has a significant effect on the corrosion behavior of the steel rebar in the concrete [8].

Nano-materials can fill the pores in the internal of concrete and enhance the compactness of concrete, which increases the protection of the steel rebar in concrete. Nano-SiO_2_ (NS) is an amorphous silica with a diameter between a few nanometers and hundred nanometers, and its particles are smaller than fly ash and cement particles, which can effectively fill pores and improve the compactness of concrete. What is more, nano-SiO_2_ has a high specific surface area and high pozzolanic activity as well as nucleation, which helps to accelerate the hydration process of cement [9,10,11]. Proper amounts of nano-SiO_2_ can increase the mechanical properties of cement concrete [12,13], improve wear resistance [14], reduce water absorption [15,16], and reduce drying shrinkage of cement concrete [17].

Fibers can inhibit crack formation [18], slowing down the steel rebar corrosion in the concrete. Polyvinyl alcohol (PVA) fibers have some advantages, such as high elastic modulus, excellent mechanical properties, acid and alkali corrosion resistance, and good compatibility with cement [19,20,21]. The addition of PVA fibers can form a three-dimensional network structure inside the concrete [22,23], effectively enhancing the toughness of cement concrete, limiting the generation and development of cracks, and increasing the flexural, compressive strength and durability of cement-based composite materials [24].

Taking advantage of the role of nano-SiO_2_ in filling and compacting concrete and the effect of PVA fiber in controlling cracks, this article investigates the hybrid effectiveness of the two materials on the corrosion behavior of steel rebar in mortar with high-volume fly ash exposed to the chloride environment. Steel rebar corrosion behavior was evaluated by using an impressed voltage technique on specimens exposed to a 5% sodium chloride solution [8]. The corrosion current, cracking behavior, and mass-loss of the steel rebar were tested, and the mechanism of performance was studied.

## 2. Materials and Methods

### 2.1. Materials

**Figure 1:**
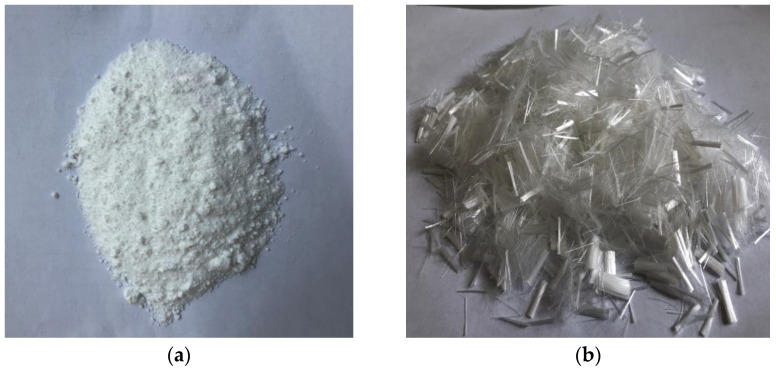
Appearance features of (**a**) NS and (**b**) PVA.

The P.O 42.5R Portland cement is output by Guangdong Tapai Cement Co., Ltd., China, and the properties are presented in Table 1. The sand is produced by Xiamen Aisiou Standard Sand Co., Ltd., China, and its fineness modulus and density are 2.85 and 2.65 g/cm^3^, respectively. The fly ash is produced by the Shouxian Power Plant in China and its properties are listed in Table 2. The main components of fly ash are Al_2_O_3_ and SiO_2_. NS is from Hangzhou Wanjing New Material Co., Ltd., China, and its diameter is about 30–50 nm, which is shown in Figure 1a. The superplasticizer is polycarboxylate superplasticizer, with water-educing efficiency of about 25%. The type of PVA fiber is K-I, which is produced by Japan Co., Ltd., Japan, and the properties are listed in Figure 1b and Table 3.

### 2.2. Specimen Preparation

The effect of NS content (0, 0.5, 1.0, and 1.5%) on the corrosion behavior of steel rebar embedded in the mortar was studied when the content of fly ash was set at 50% and 60%, and the content of PVA was fixed at 1.0% by volume. The mix proportions are shown in Table 4.

**Figure 2:**
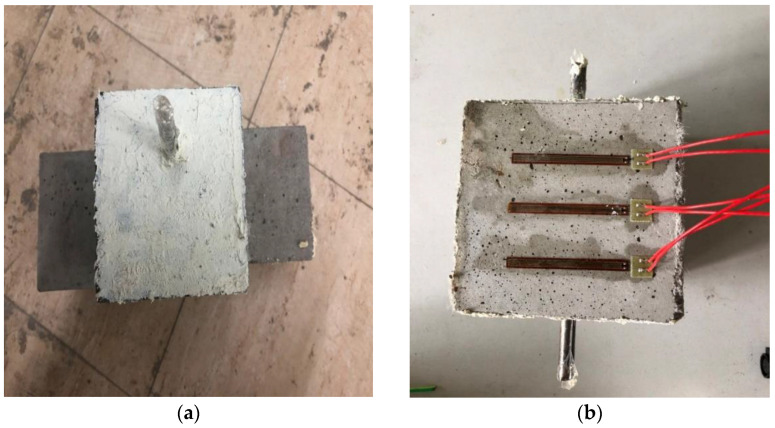
Test specimen (**a**) the side coated by epoxy, (**b**) 3-strain gauges on the surface.

The specimens of the modified mortar were prepared by the following experimental steps [25]:

(1) Weighing the cement, sand, FA, NS, PVA fiber, water, and superplasticizer;
(2) Dry-mixing the sand, cement, NS, FA, and superplasticizer for 2 min;
(3) Adding water to the dry mixture and continuing to mix for 2 min;
(4) Adding PVA fiber to the mixture with a certain fluidity along the stirring direction, and then stirring for 3 min;
(5) Pouring the mixed mortar into molds prepared with pre-embedded steel rebar and ensuring good compaction;
(6) Smoothing the surface of the specimens and wrapping the exposed steel rebar to prevent corrosion;
(7) Demolding the specimens after 24 h and then curing them for 28 days in the curing room;
(8) Two days before the test, coating both sides of the exposed steel rebars with epoxy resin to prevent the solution from contacting the steel rebar through the surface (preventing “neck brittleness” damage, as shown in Figure 2a);
(9) Pasting 3-strain gauges on the surface, 25 mm away from the steel rebar (as shown in Figure 2b).

### 2.3. Experimental Methods

#### 2.3.1. Mechanical Performance Test

The flexural and compressive strength of the specimens were tested according to the “Test Method for Strength of Cement Mortar Sand” (GB/T 17671-1999) [26]. For each mix in Table 4, three specimens of 160 mm × 40 mm × 40 mm in dimensions were tested by three-point bending with supporting span of 100 mm, and six 100 mm × 100 mm × 100 mm cubic specimens were used for compressive strength tests. The automatic machine CMT-5105 was used to flexural strength test. The test measure is in displacement control mode with a loading rate of 0.05 mm/min. A servo-hydraulic YAW-4306 testing system was utilized to test the compressive strength with a loading rate of 0.5 MPa/s.

#### 2.3.2. Water Absorption Test

**Figure 3:**
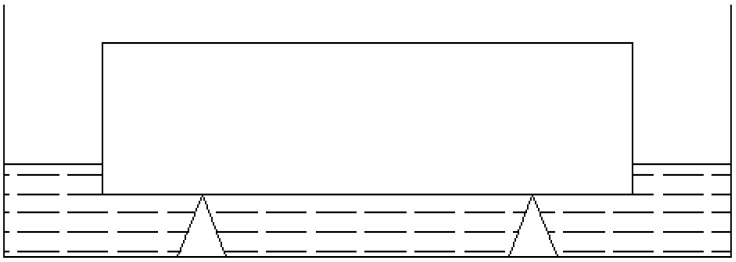
Capillary water-absorption test device.

**Figure 4:**
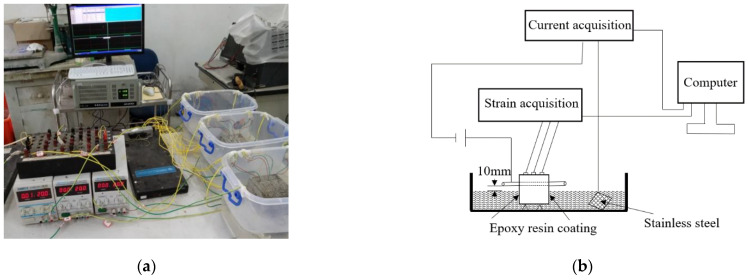
Test equipment for accelerated corrosion (**a**) test equipment, (**b**) schematic diagram of the test system.

Specimens (40 mm × 40 mm × 160 mm) were cured for 28 days in the curing room. Specimens were placed in blast air and not taken out unless their mass loss rate was below 0.1% within 24 h at the temperature of 50 °C. After drying, the specimens were taken out and weighed. The four sides of the specimens were sealed with paraffin, and two sides of 40 mm × 160 mm were reserved to make sure that the process of capillary water absorption was one-dimensional. The water absorption test followed these steps [27]: (1) put the support in a flat-bottomed container; (2) put the cement mortar on the support with the side of the specimen not coated with paraffin facing down; (3) slowly pour clear water into the container until the water surface is 5 mm higher than the bottom of the specimen (as shown in Figure 3); (4) take out the specimens at different accumulative times (0 min, 5 min, 10 min, 20 min, 30 min, 60 min, 120 min, 180 min, 240 min), quickly wipe off the surface moisture, and then weigh and record their weight; (5) the unit water absorption of the specimen can be calculated by Equation (1), and the capillary water-absorption coefficient of the specimen can be calculated from the slope.

(1) $$\frac{Q}{A}=k\sqrt{t}$$

where *Q* is water absorption mass of the specimen (g), *A* is the surface area of the water absorption surface of the specimen (m^2^), *t* is the cumulative water absorption time (min), and *k* is the capillary water-absorption coefficient of the specimen (g/(m^2^·min^1/2^)).

#### 2.3.3. Impressed Voltage Accelerated Corrosion Test

The experiment with the impressed voltage corrosion test was implemented on mortar specimens containing a single piece of steel rebar, and the specimens and equipment for the accelerated corrosion test are shown in Figure 2 and Figure 4. So as to accelerate the corrosion process of steel rebar in a short time, an external direct current source with a constant voltage of 20 V was used in the system during testing. The devices and schematic of the test are shown in Figure 4a,b. In the experiment, the steel rebar acts as the anode, and the stainless-steel mesh acts as the cathode. During the experiment, the cracking process of specimen surface was recorded with strain gauges, and the impressed current was also recorded by current meter. The accelerated corrosion experiment was stopped when the width of a surface crack was 1 mm. After the test, the rust distribution in the mortar was observed by breaking the specimens. The steel rebar was taken out from the specimen and cleaned, and then its mass was measured and recorded. The mass loss ΔM and the corrosion level *ρ* were determined by the following Equations (2) and (3):

(2) $$∆M=\frac{M_{0}-M_{1}}{M_{0}}\times 100\%$$

(3) $$\rho =\frac{∆M_{i}}{∆M_{c}}\times 100\%$$

where $M_{0}$ is the steel rebar’s original mass, $M_{1}$ is the cleaned rebar’s mass after corrosion experiment, $∆M_{i}$ is the mass loss of the steel rebar embedded in the modified mortar, and $∆M_{c}$ is the mass loss of the steel rebar embedded in the control mortar.

## 3. Results and Discussion

### 3.1. Mechanical Performance

**Figure 5:**
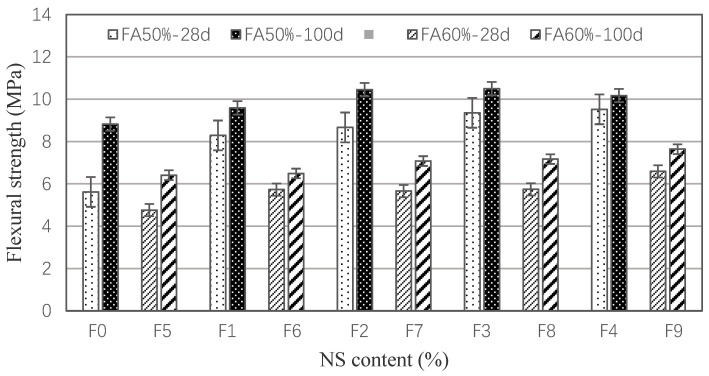
Flexural strength of NS-modified mortar.

The influence of the different contents of NS on the flexural strengths of 50% and 60% fly ash mortar is shown in Figure 5. The 28-day flexural strength of all the NS-modified mortars is higher than that of the control mortar, which indicates NS can enhance the strength of mortar [28], and as the NS content increased, the 28-day flexural strength also increased, which is similar to the results in the literature [29]. Compared to the common 50% fly ash mortar, the 28-day flexural strength of F2–F4 mortar is increased by 54.3–69.4%. The incorporation of 1.0% PVA fiber alone can also improve the flexural strength, which is 47.5% and 8.73% higher than that of the control group by 28 and 100 days, respectively. For the 60% fly ash mortar, the flexural strength is lower than that of 50% fly ash mortar. Also, for the group of F7–F9, the flexural strength is 18.9–38.5% higher than the F5. The F6 mortar is 20.4% higher than the control mortar. When the curing time is 100 days, the flexural strength of 50% and 60% fly ash is 56.9% and 34.7% higher than that of their 28-day-cured counterparts, respectively. In Figure 5, we can also find that the more fly ash that is added, the lower the flexural strength is. What is more, the 28-day flexural strength of 50% fly ash mortar for F2–F4 are 8.67–9.52 MPa, which are higher than the 100-day strength of 60% fly ash mortar for F7–F9.

**Figure 6:**
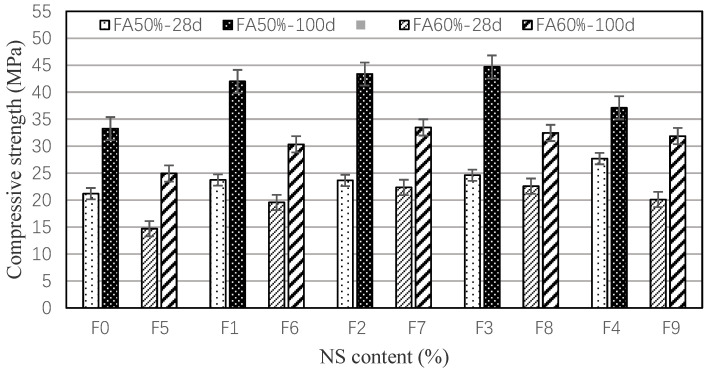
Compressive strength of NS-modified mortar.

Compressive strength results of modified mortar with different contents of NS are shown in Figure 6. The compressive strength of NS-modified mortar is generally enhanced. As to the 28-day compressive strength of 50% fly ash mortar, the larger the NS content, the greater the strength increase, and the compressive strength of F4 is 30.7% higher than control mortar, with the PVA-only mortar exhibiting an enhancement of 11.9% in compressive strength. For the 100-day curing, with the incorporation of NS increasing, the compressive strength increases first and then decreases. The compressive strength of F3 mortar is 44.68 MPa, which is 34.4% higher than the control mortar. When compared with the 28-day mortar, the compressive strength of all 100-day mortar is increased. The 100-day strength of F2–F4 mortar is 83.5–34.0% higher than that of 28-day mortar. For the 60% fly ash mortar, the enhanced compressive strength trend is the same as for 50% fly ash mortar. For the group of F7–F9, the 28-day compressive strength is 36.7–53.6% higher than the F5 mortar, and the 100-day is 27.8–34.2% higher. NS can accelerate the hydration of FA-cement systems [10,30], so the compressive strength is improved.

### 3.2. Water Absorption

**Figure 7:**
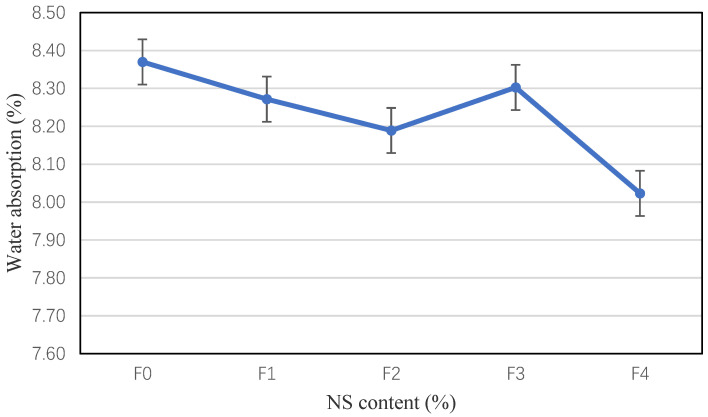
Water absorption of NS-modified mortar.

Figure 7 shows the results of the water absorption experiments. It is clear that the water absorption of the mortar decreases with the incorporation of NS. The water absorption of F1 modified mortar is 1.2% lower than control mortar, and the water absorption of F2–F4 is 1.0–4.2% lower than control mortar. NS can fill bubbles and reduce the porosity of mortar, which can enhance the compactness and reduce water absorption.

**Figure 8:**
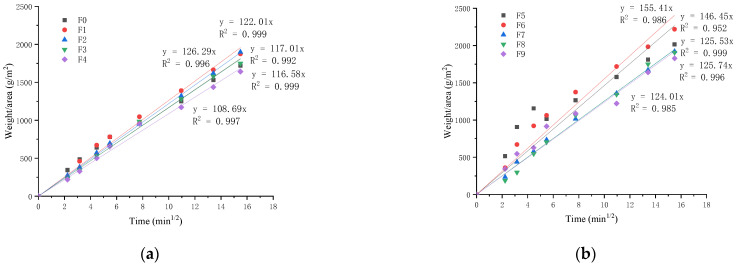
Capillary water absorption of NS-modified mortar: (**a**) 50% FA and (**b**) 60% FA.

Figure 8 shows the effect of NS on the mortar’s capillary water absorption. The fly ash content is 50% in Figure 8a. The capillary water absorption of the PVA-only modified mortar is obviously increased because of the air-entraining effect of the PVA fiber. The capillary water absorption coefficient of the F1 mortar reaches 126 g/(m^2^·min^1/2^), which is greater than the control mortar’s 117 g/(m^2^·min^1/2^). PVA fibers can increase the holes in mortar and make them communicate with each other, which is the reason for the increase in the capillary water absorption coefficient. The incorporation of NS significantly reduces the capillary water absorption of the PVA specimens. For the group of F2, F3 and F4, the coefficient of capillary water absorption is 122 g/(m^2^·min^1/2^), 116 g/(m^2^·min^1/2^), and 108 g/(m^2^·min^1/2^), respectively. NS can fill pores and reduce the internal pores, so it can reduce the capillary water absorption of the modified mortar.

When the fly ash is 60%, the result of capillary water absorption is shown in Figure 8b. It is obvious that the coefficient of 60% FA-mortar capillary water-absorption is much larger than that of 50% FA mortar. That means the greater the fly ash addition, the larger the coefficient is. For the group of F7, F8, and F9, the coefficient of capillary water absorption is 125 g/(m^2^·min^1/2^), 125 g/(m^2^·min^1/2^), and 124 g/(m^2^·min^1/2^), which is much lower than the control mortar of 146. As with 50% FA mortar, the coefficient of PVA-only modified mortar is the largest.

### 3.3. Test Result of Accelerated Corrosion

#### 3.3.1. Impressed Current

**Figure 9:**
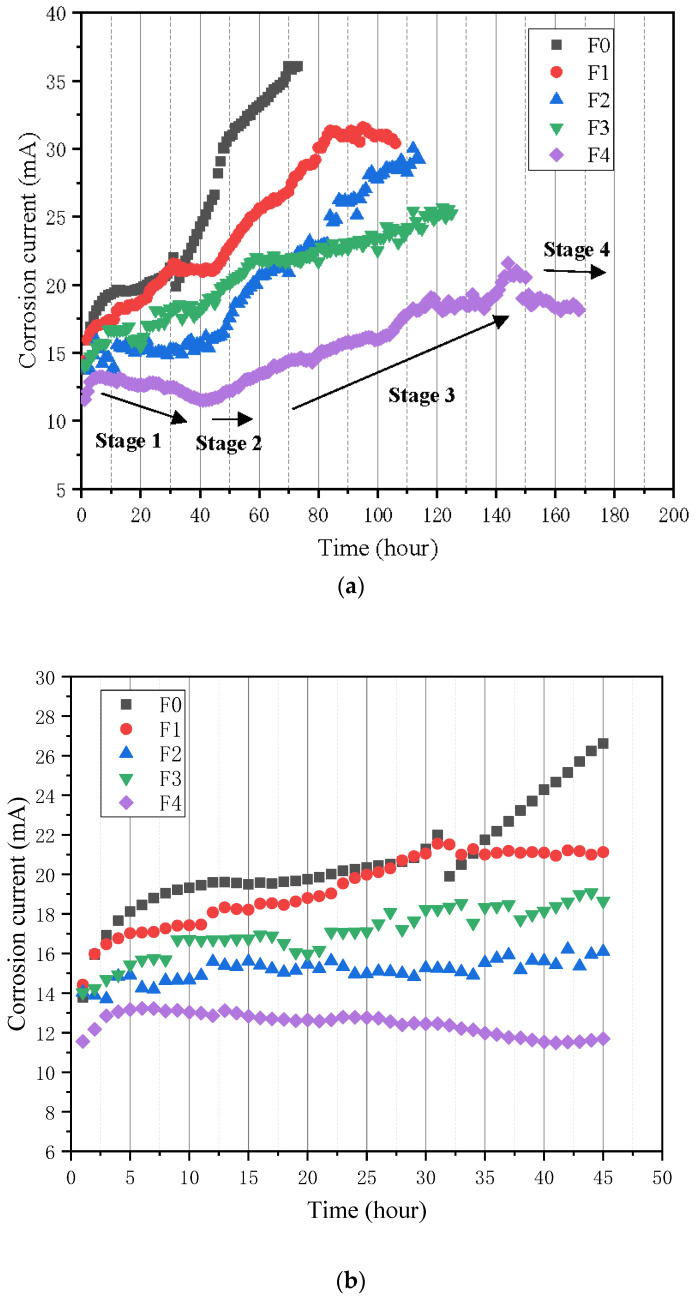
Corrosion current of 50% FA mortar: (**a**) the whole corrosion time and (**b**) the first 40 h corrosion time.

The impressed currents of 50 wt.% fly ash cement mortars under the accelerated corrosion are shown in Figure 9a, and Figure 9b shows the enlargement of the corrosion current at the first 45 h; it can be found in this figure that the F0 had the highest current, followed by F1, and F4 had the lowest current. The corrosion process of steel rebar in mortar is essentially an electrochemical process [31,32,33]. The impressed current is greatly affected by the ionic conductivity of cement mortar, which is determined by the porosity, tortuosity and connectivity of the matrix. Generally, the higher the porosity, the more the intercommunicating between the pores, the higher ionic conductivity, and the larger impressed current will be [34]. It can be seen in Figure 9b that the initial currents of F0, F1, F2, F3, and F4 are 15.9 mA, 16.0 mA, 13.9 mA, 14.2 mA, and 12.2 mA, respectively. The initial current of F1 is almost the same as that of F0, which is mainly because of the connectivity of the pore structure of the mortar incorporating the PVA fibers. However, the initial current of the mortar modified by the hybrid addition of NS and PVA is reduced, and the initial current of F4 is only 12.2 mA, which is significantly smaller than that of the control mortar with an impressed current of 15.9 mA.

The corrosion current curves can be considered as four stages in Figure 9a: (1) a descending phase, (2) a steady phase, (3) an ascending phase, and (4) another steady phase. In the first stage, the corrosion productions of the iron-ion oxidation reaction gradually reduce the porosity of the steel rebar and the mortar before the generation of cracks, which can hinder the ingress of air and free ions. Therefore, the impressed current of mortar decreases. In the second stage, the porosity of the interface between the steel rebar and the mortar drops to a certain level, so a balance is established between the transmission and consumption rates of oxygen and free ions. In this stage, the impressed current is stable. In the third stage, microcracks generate and expand at the interface of the reinforced mortar due to the expansion force caused by corrosion, resulting in the increase of the intrusion speed of the oxygen and iron ions. Therefore, at this stage, the corrosion rate increases as the corrosion time increases. Additionally, the products of corrosion diffuse along the cracks and fill the pores of the mortar. In the fourth stage, continuous longitudinal cracks form in the mortar, and at the same time, oxygen accesses the steel rebar at a steady speed. In this case, the rate of corrosion reaches its maximum and tends to be steady. The impressed current of F0 and F1 do not decrease in the first stage but increase. The main reason is that the generated rust products are quickly transferred to the pores without affecting the accelerated corrosion process, and the external oxygen enters quickly, so the impressed current in the first stage slowly increases.

From the above mechanism we can know that the second turning point (from stage 2 to 3, in Figure 9a) indicates the time (t_ini_) of the microcracks caused by corrosion in the interface between steel and mortar. The third turning point (from stage 3 to 4, in Figure 9a) represents the forming continuous longitudinal crack time (t_cr_) in the mortar. It is obvious that t_ini_ and t_cr_ are closely connected with the corrosion level of the steel rebar: in general, the lower the corrosion level of the steel rebar is, the higher values of t_ini_ and t_cr_ are, and the corrosion products fill the pores of the mortar before the forming of micro and continuous longitudinal cracks inside the mortar, so a higher porosity may also result in bigger t_ini_ and t_cr_. All the specimens’ t_ini_ and t_cr_ values during the measurement process are shown in Table 5. In this table, all the composite incorporation of PVA and NS can delay the time of forming of the interfacial cracks between the steel rebar and mortar interface and the time of the forming of the continuous longitudinal cracks during the corrosion process. For the group of F4, the t_ini_ value is 51 h, which is almost 1.6 times that of the control group. The time t_cr_ of forming continuous longitudinal cracks is 144 h, which is 2 times that of the control group. The t_ini_ and t_cr_ values of the F1 modified mortar are 1.4 times and 1.2 times higher, respectively, than those of the unmixed group.

#### 3.3.2. Cracking Characteristics of Mortar Cover 

**Figure 10:**
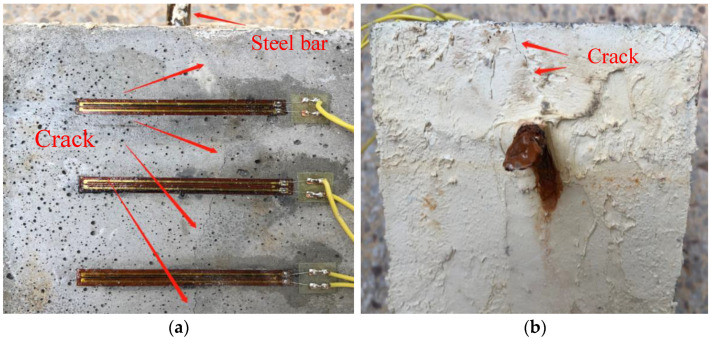
Cracking characteristics on the mortar cover during the corrosion experiment (**a**) crack on the gauges side, (**b**) crack on the exposed steel rebar side.

**Figure 11:**
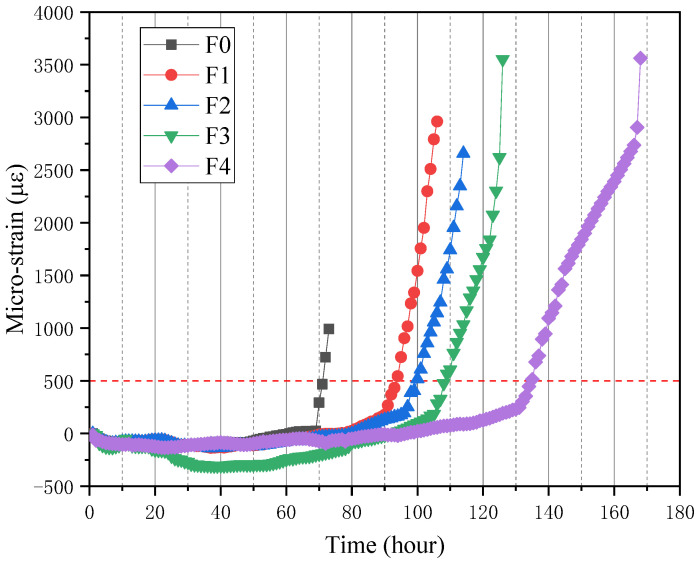
Micro-strain of 50% content FA specimen.

Figure 10 shows the results of cracking characteristics of mortar during the corrosion experiment. Obviously, the cracks of mortar appear on the upper side and parallel to the steel rebar. Therefore, so as to quantitatively study the effect of NS and PVA on the cracking characteristics of the mortar cover, the relationship between the strain of specimen and the corrosion time was recorded by strain gauges, and the results are shown in Figure 11.

Table 5 and Figure 11 show the average time (t_visi_) of the visible cracks on the surface of all specimens. According to the test results, the single-mixed PVA mortar can prolong the corrosion damage time of the mortar cover, and the t_visi_ value of the F1 specimen is 106 h, which is 45% higher than that of the unmixed mortar. That is because PVA fiber can control the cracks. On the other hand, all the specimens mixed with PVA and NS can reduce the deterioration rate of the mortar cover. For the group of F2, F3, and F4, the t_visi_ values are 56%, 71% and 130% higher than F0, respectively. In a word, the hybrid additions of NS and PVA delayed the generation of visible cracks in the specimens and also improved the durability of the mortar.

#### 3.3.3. Mass Loss of Steel Rebar and Rust Distribution

The mass loss (Equation (2)) and corrosion levels (Equation (3)) for all the specimens are calculated. The results of mass loss and corrosion levels are shown in Table 5, and the corrosion level of the steel rebar in F0 is treated as the evaluation index of 100%. The mass loss of the steel rebars of the F1 is higher than the control mortar, and the corrosion level is 12% higher than the control mortar. The hybridization of PVA and NS can reduce the corrosion level of the steel rebar, and for the group of F2, F3, and F4, the corrosion level is reduced by 30%, 49%, and 19%, respectively, compared to the control mortar. These results once again confirmed that the incorporation of NS and PVA has a positive effect on the corrosion resistance of the steel rebar in the mortar.

**Figure 12:**
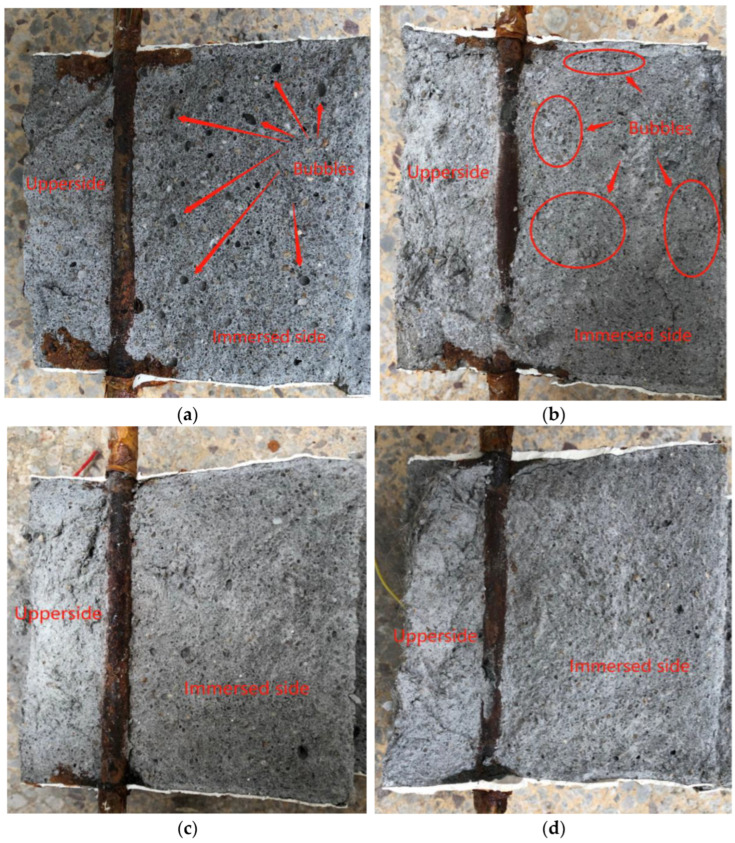
Distribution of rust in the splitting mortar: (**a**) FA; (**b**) PVA; (**c**) NS1.0; (**d**) NS1.5.

Figure 12 shows the distribution of corrosion products in the mortar cover after splitting. It can be found that the corrosion products in the upper side are usually more than those in the bottom side of the specimen. Researchers such as Angst [6] found a similar result and conducted a detailed analysis. The uneven distribution of rust within mortar may be due to two factors: one is that the corrosion production diffuses mainly along the cracks and fills the pores around the mortar; the other is that the volume expansion caused by the rebar corrosion induces the upper side mortar’s cracking. What is more, in the figure, it can be found that the incorporation of NS and PVA significantly reduces the content of the corrosion products in the reinforced mortar. As shown in Figure 12a, the corrosion products start from the surface of the steel rebar and extend to the surface of the specimen. These are full of rust along the interface between the steel rebar and the mortar. In addition, it can be found that there are many large bubbles in the mortar (Figure 12a). In Figure 12b, the rust is much reduced. The main reason is that the mass loss of the steel rebar of the single-doped PVA specimens is lower, so there is less rust. Moreover, the diameter of the bubbles in Figure 12b is small, and the small pore size indicates that the PVA’s workability during mixing and vibration refines the pore size inside the mortar, turning a large diameter hole into a small diameter bubble. It is found in Figure 12c,d that there is less rust in the mortar, mainly because NS can fill and refine the pores and improve the microstructure of the mortar, reduce the impressed current, and the mass loss of the steel rebar. All in all, NS plays a positive role in the protection of the steel rebar.

## 4. Conclusions

This article mainly studied the hybrid effects of NS and PVA on the mechanical properties of mortar, capillary water absorption, the and corrosion of steel rebar embedded in cement mortar under accelerated chloride erosion. In the light of the experimental results and theoretical analysis, the following conclusions were drawn:

1. The hybrid incorporation of PVA and NS can obviously improve the mechanical properties of large-volume fly ash mortar. For the 50% FA mortar, the flexural and compressive strength of single-mixed PVA mortar increased by 47.5% and 11.9%, respectively, compared to the control mortar, and the greater the incorporation of NS content, the higher the flexural and compressive strength of cement mortar. When containing 1.0 vol.% PVA fibers, the incorporating of 0.5–1.5% NS enhanced the 28-days flexural and compressive strengths by 54.3–69.4% and 11.6–30.7%, respectively. Moreover, the incorporation of NS was effective in improving the strength development of the cement mortar, and the 100-days strength of 0.5–1.5% NS-modified mortar was about 34.0–83.5% higher than that of the 28-day strength. For the 60% fly ash mortar, PVA and NS can also enhance the mechanical properties of modified mortar, and the trend is similar to that with 50% fly ash. The flexural and compressive strengths of cement mortars with 50% FA were generally higher than those of mortars with 60% FA.

2. PVA-alone increases the capillary water absorption of the modified mortar. The hybrid incorporation of NS and PVA can reduce the capillary water absorption. The coefficient of capillary water absorption of the 50% FA mortar was 126 g/(m^2^·min^1/2^), which was smaller than 60% FA mortar with a coefficient of 146 g/(m^2^·min^1/2^). When the PVA content is 1.0% and NS content is 0.5%, 1.0%, and 1.5%, the coefficient of water absorption for 50% FA mortar is 122 g/(m^2^·min^1/2^), 116 g/(m^2^·min^1/2^), and 108 g/(m^2^·min^1/2^), respectively, and for the 60% FA mortar, the coefficient incorporating of 0.5%, 1.0%, and 1.5% NS, the coefficient is about 125 g/(m^2^·min^1/2^), 125 g/(m^2^·min^1/2^), and 124 g/(m^2^·min^1/2^), respectively.

3. The incorporating of NS and PVA enhances the resistance to chloride-ion erosion for cement mortar effectively. When the specimen is immersed in 5% NaCl solution, the incorporation of NS and PVA greatly reduces the corrosion current and rust in mortar. When hybrid-incorporating 1.0 vol.% PVA and 1.0 wt.% nano-SiO_2_, steel rebar had the lowest corrosion degree, with a mass loss of 49%, and the broken time increased by 71% as compared to the control mortar.

## Figures and Tables

**Table 1:** Physical and chemical properties of cement.

Mass Fraction/%								Setting Time/min		*F*_t_/MPa		*F*_c_/MPa	
SiO_2_	Al_2_O_3_	CaO	MgO	Na_2_O	K_2_O	Fe_2_O_3_	SO_3_	Initial setting	Final setting	3 d	28 d	3 d	28 d
18.3	4.5	62.4	2.1	0.3	1.5	2.3	3.5	72	180	5.5	8.6	28.0	50.6

*F*_t_ is tensile strength; *F*_c_ is compressive strength.

**Table 2:** Chemical properties of fly ash.

	Al_2_O_3_	SiO_2_	SO_4_	K_2_O	CaO	TiO_2_	MnO	Fe_2_O_3_
FA (%)	37.3540	48.9115	1.1817	0.8178	5.4984	1.8097	0.0291	4.3979

**Table 3:** The main characteristics of PVA.

Diameter/mm	Length/mm	Density/g·cm^−3^	Tensile Strength/MPa	Elastic Modulus/GPa	Elongation/%	Aspect Ratio
0.038	12	1.3	1092	30	7	316

**Table 4:** Mix ratio of high-volume fly ash mortar.

Material	Mix Proportions/(kg·m^–3^)				Nano-SiO_2_ (wt)/%	PVA Fiber (vol)/%	Superplasticizer (wt)/%
	Water	Cement	Fly Ash	Sand			
F0	400	500	500	1100	0	0	1.0
F1	400	500	500	1100	0	1.0	1.0
F2	400	500	500	1100	0.5	1.0	1.0
F3	400	500	500	1100	1.0	1.0	1.0
F4	400	500	500	1100	1.5	1.0	1.0
F5	400	400	600	1100	0	0	1.0
F6	400	400	600	1100	0	1.0	1.0
F7	400	400	600	1100	0.5	1.0	1.0
F8	400	400	600	1100	1.0	1.0	1.0
F9	400	400	600	1100	1.5	1.0	1.0

**Table 5:** Corrosion characteristics of reinforced mortar.

	Time of Forming Microcracks on Rebar/Mortar Interface, t_ini_ (h)	Time of Forming Continuous Cracks inMortar Cover, t_cr_ (h)	Time of Forming Visible Cracks on Mortar Surface, t_visi_ (h)	Mass Loss of Rebar (%)	Corrosion Level, *ρ* (%)
F0	32	71	73	2.93	100
F1	44	87	106	3.27	112
F2	43	109	114	2.04	70
F3	50	119	125	1.49	51
F4	51	144	168	2.37	81

## Data Availability

The data used to support the findings of this study are available from the corresponding author upon request.
